# Thromboembolic complications of *Mycoplasma pneumoniae* pneumonia in children

**DOI:** 10.1111/crj.13584

**Published:** 2023-01-19

**Authors:** Lanqin Chen, Ju Yin, Xiuyun Liu, Jun Liu, Baoping Xu, Kunling Shen

**Affiliations:** ^1^ Department of Respiratory Diseases I Beijing Children's Hospital, Capital Medical University, National Clinical Research Center for Respiratory Diseases, National Center for Children's Health Beijing China

**Keywords:** children, *Mycoplasma pneumoniae*, pneumonia, thromboembolism

## Abstract

**Background:**

Thromboembolism is less common in children than in adults, but it is frequently associated with *Mycoplasma pneumoniae* infection in many cases. This study aimed to investigate the clinical characteristics of pediatric *M. pneumoniae* pneumonia complicated with thromboembolism.

**Methods:**

Hospitalized patients with *M. pneumoniae* pneumonia complicated by thromboembolism were enrolled from January 2012 to December 2021 in Beijing Children's Hospital, Capital Medical University, China. The data on clinical manifestations, laboratory tests, and treatment were evaluated.

**Results:**

A total of 49 cases were enrolled, with a mean age of 7.9 years old, including 27 boys and 22 girls. Consolidation of pulmonary lobe or segment was observed in 95.9% (47/49) of the cases, whereas interstitial change was found only in two patients; 85.7% (42/49) of patients had pleural effusion. Pulmonary vascular thromboembolism was most common in 35 patients, whereas 13 cases had thromboembolism of multiple anatomic sites. The levels of C‐reaction protein, lactate dehydrogenase, and erythrocyte sedimentation rate were all increased, with a mean value of 54.08 ± 52.27 g/L, 451.12 ± 218.76 U/L, 43.40 ± 29.43 mm/h, respectively. Blood coagulation test showed that all 49 patients had elevated D‐dimer values (median 3.81 ng/ml, range, 0.34–48 ng/ml) and normal PT. aPTT.LA was positive in 74.3% (26/35) of the cases. aCL‐IgM was positive in 66.7% (26/39) of the cases. aβ2GPI‐IgM was positive in 79.4% (27/34) of the cases. The prognosis was generally good in this group.

**Conclusion:**

Pulmonary arteriovenous thromboembolism is the most common thromboembolism complicated in MPP, and cerebral artery embolism and cardiac thrombosis are common in extrapulmonary thromboembolism. In the cases of MPP with thromboembolic complications, pulmonary consolidation with pleural effusion is the main characteristic. About two thirds of the cases are positive for antiphospholipid antibodies.

## Graphical abstract

A retrospective study to investigate the clinical characteristics of pediatric *mycoplasma pneumoniae* pneumonia complicated with thromboembolism.

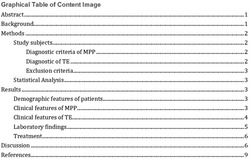

**Abbreviations**
DVT: deep venous thrombosis
LMWH: lower molecular weight heparin
MCA: middle cerebral artery
MPP: *Mycoplasma pneumoniae* pneumonia
PE: pulmonary artery embolism
RV: right ventricle
SMA: arterial mesenterica superior

## 1 BACKGROUND

Infection with *Mycoplasma pneumoniae* causes 10–40% of community‐acquired pneumonia cases in children and 18% of cases requiring hospitalization,^1^, ^2^ with these percentages increasing during epidemics. *M. pneumoniae* pneumonia (MPP) and severe MPP have increased in China.^3^ MPP leads to numerous extra‐respiratory manifestations of variable severity, including dermatological manifestations and neurological complications, whereas thromboembolism is a rare but severe complication.^4^ Several reports about thrombosis or embolism are complicated with MPP, mainly case reports.^5^, ^6^ Herein, we review a case series of thromboembolic complications in MPP.

## 2 METHODS

### 2.1 Study subjects

We retrospectively reviewed hospitalized patients under 18 years old with MPP and thromboembolism and treated them between January 2012 and December 2021 in Beijing Children's Hospital, Capital Medical University, China.

Collected information included the following: (1) Demographic features of the patients, course of the disease, and past medical history; (2) clinical features and symptoms; (3) laboratory tests, including pathogen tests, imaging, ultrasounds, and CT; and (4) treatment and prognosis. Information on the prognosis of pediatric patients was collected using telephonic follow‐up.

#### 2.1.1 Diagnostic criteria of MPP

In this study, the diagnosis of MPP met the following standards: (1) Acute respiratory symptoms (cough, fever, dry or productive sputum, dyspnea) in previously healthy children; (2) presence of abnormal chest radiography or abnormal lung sound, and (3) *M. pneumoniae* infection is defined as single titers of serum *M. pneumoniae* antibody ≥1:320, or single titers of serum *M. pneumoniae* antibody ≥1:160 with positive PCR of *M. pneumoniae* or seroconversion (antibody titer increase of more than or equal to fourfold between paired sera).^7^

#### 2.1.2 Diagnostic of thromboembolism

The diagnosis of thromboembolism was confirmed by various imaging techniques, including Doppler ultrasound, CT, and magnetic resonance imaging (MRI), or pathology.

A pulmonary embolism (PE) was diagnosed by ventilation‐perfusion lung scan, enhanced CT scan, or pulmonary angiography. Deep vein thrombosis (DVT) and the presence of thromboses in renal, portal, and mesenterial veins were confirmed by ultrasonography, venography, CT scan, or MRI findings. Arterial embolism was confirmed by contrast angiography, Doppler ULS, CT scan, MRI, or surgery.

Radiographic examinations were anonymized and reviewed by two radiologists to remove bias associated with them.

#### 2.1.3 Exclusion criteria

Patients with tumors, previously diagnosed autoimmune diseases (such as Takayasu arteritis and antiphospholipid syndrome), immunodeficiency diseases, and congenital vascular malformations (Moyamoya disease, etc.) were excluded.

This study was performed in compliance with the Helsinki Declaration. The Ethical Principles for Medical Research Involving Human Subjects were approved by the Ethics Committee at Beijing Children's Hospital, Capital University of Medical Science. During admission, informed consent was obtained for the patients' clinical records in future studies. The data were accessed anonymously and analyzed statistically.

### 2.2 Statistical analysis

Continuous variables were described by means and standard deviations, or ranges, based on the normality of data. Frequencies or percentages were described as categorical variables. The Kolmogorov–Smirnov test evaluated the normality of data in each group. Continuous variables were analyzed by an independent sample *t*‐test or Mann–Whitney *U*‐test, based on the normality of the data. Categorical variables were analyzed by the *χ* ^2^‐test. Multivariate logistic regression was used to identify the influencing factors of therapeutic efficacy. We used SPSS (version 19.0) for all analyses, and a *P* value <0.05 was considered significant.

## 3 RESULTS

### 3.1 Demographic features of patients

Forty‐nine cases of complex thromboembolism in MPP were enrolled in this study, comprising 27 boys and 22 girls, for a male‐to‐female ratio of nearly 1.23:1. The mean onset age was 7.90 ± 2.72 years old, whereas it was most common in the age group of 5–12 years old (*n* = 39, 79.6%). All the patients in this group were healthy in the past and had no history of drug use, including oral contraceptives. All the cases denied a thromboembolism history of first‐degree relatives. Five patients had a history of bronchoscopy surgery under general anesthesia before the onset of thromboembolism. The clinical characteristics and laboratory values of MPP patients with thromboembolism were listed in Table 1.

**TABLE 1:** Clinical characteristics and laboratory values of MPP patients with thromboembolism

	Total (*n* = 49)
Gender, male/female%	27/22 (55)
Age, *n* (%)	
1–4 years	6 (12.2)
5–12 years	39 (79.6)
12–18 years	4 (8.2)
Clinical factors, *n* (%)	5 (10.2)
Bronchoscopy surgery under general anesthesia	
Image of pneumonia, *n* (%)	
Consolidation	47 (95.9)
Pleural effusion	42 (85.7)
Necrotizing pneumonia	29 (59.2)
D‐dimer, *n* (%)	
>5 μg/ml	21 (42.8)
<5 μg/ml	28 (57.2)
Laboratory finding	Mean (SD, range)
WBC (k/μl)	11.40 (4.33, 4.88–24.14)
Hemoglobin (g/dl)	115.12 (16.11)
Platelets (k/μl)	319.65 (145.91)
CRP (mg/L)	54.08 (52.27)
ESR (mm/h)	43.40 (29.43)
LDH(U/L)	451.12 (218.76)
D‐dimer (μg/ml)	3.81 (range, 0.34–48)
PT (s)	12.79 (1.75)
aPTT (s)	32.43 (6.34)
Fibrinogen (mg/dl)	3.81 (1.18)
Antiphospholipid (aPL) antibodies	*n*/*N* (%)
Lupus anticoagulant	26/35 (74.3)
Anti‐cardiolipin (aCL) antibodies	
IgM	26/39 (66.7)
IgG	4/39 (10.2)
Anti‐beta2 glycoprotein I (aβ2GPI) antibodies	
IgM	27/34 (79.4)
IgG	0/34 (0)
Ig A	2/34 (5.9)

### 3.2 Clinical features of MPP

All forty‐nine patients presented with a fever and cough. The duration between the symptom onset of MPP and thromboembolism was 11.8 days (range, 4.0–24.0 days).

The antibody titer of *M. pneumoniae* in all patients was ≥1:320 and an *M. pneumoniae* PCR test was detected in 39 cases; 26 cases were positive, and 10 cases were negative. The positive rate of PCR was significantly correlated with the course of the disease. Tests of macrolide resistance were performed only in five cases, with three positives and two negatives. On the initial chest radiograph at the hospitalization time, consolidation of the pulmonary lobe or segment was observed in 95.9% (47/49) of the cases, whereas interstitial change was found only in two patients. 85.7% (42/49) of the patients had pleural effusion. Necrotizing pneumonia (NP) was revealed in 59.2% (29/49) of the cases at the recovery stage.

### 3.3 Clinical features of thromboembolism

**CHART 1:**
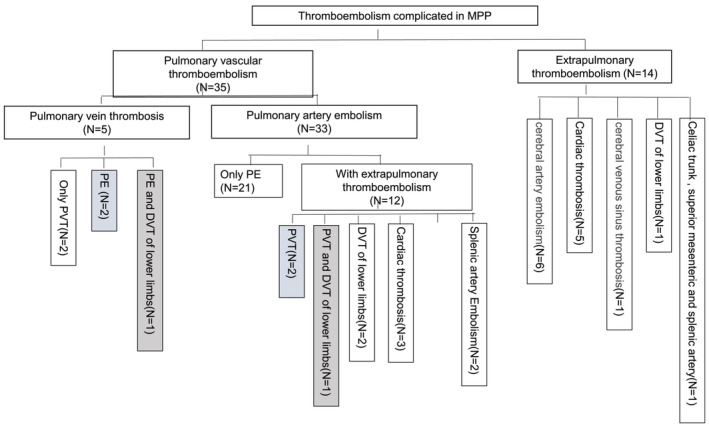
The sites of TE in MPP, grouped by pulmonary vascular and extrapulmonary vascular involved

Both arterial embolism and venous thrombosis occurred in this group, as seen in Table 2 and Chart 1.

**TABLE 2:** Sites of thromboembolism complicated in MPP

Sites of TE	Number of cases (*n*)	Percentage (%)
Pulmonary vascular	38	77.5
Pulmonary artery	33	67.3
Pulmonary vein	5	10.2
Heart	8	16.3
Ventricle	6	
Left ventricle	1	
Right ventricle	5	
Atrium	2	
Cerebrovascular	9	18.4
Middle cerebral artery	5	
Right	4	
Left	1	
Anterior cerebral artery	1	
Cerebral sinus venous	3	
DVT of lower limbs	4	8.2
Unilateral limb	3	
Bilateral lower limbs	1	
Splenic artery	4	8.2
Celiac trunk artery and Superior mesenteric artery	1	0.02

Pulmonary artery embolism (PE) was most common in 33 of 49 patients. In the PE cases, 45.5% (15/33) had symptoms including chest pain (10/15), dyspnea (6/15), hemoptysis (3/15), and chest distress (3/15), whereas 54.5%(18 of 33) cases were asymptomatic and asymptomatic PE were revealed by CTPA due to the presence of DVT. Thrombi were located in subsegmental pulmonary arteries and distal branches in 14 cases (42.4%), the segmental pulmonary arteries in nine cases (27.2%), the lobar pulmonary artery in nine cases (24.2%), and the left pulmonary trunk in two cases. In 69.7% (23/33) of the cases, the embolism was within the consolidation of pneumonia, whereas in 30.3% of cases, the embolism was outside of the consolidation. There were 29 cases with NP. The majority of NP cases (82.8%, 24/29) were complicated with PE, whereas in the non‐NP group, the number of cases complicated (*n* = 9 vs. 11) or uncomplicated (*n* = 9 vs. 11)were nearly equal. PE is associated with NP (*χ* ^2^ = 7.674, *P* = 0.006).

**FIGURE 1:**
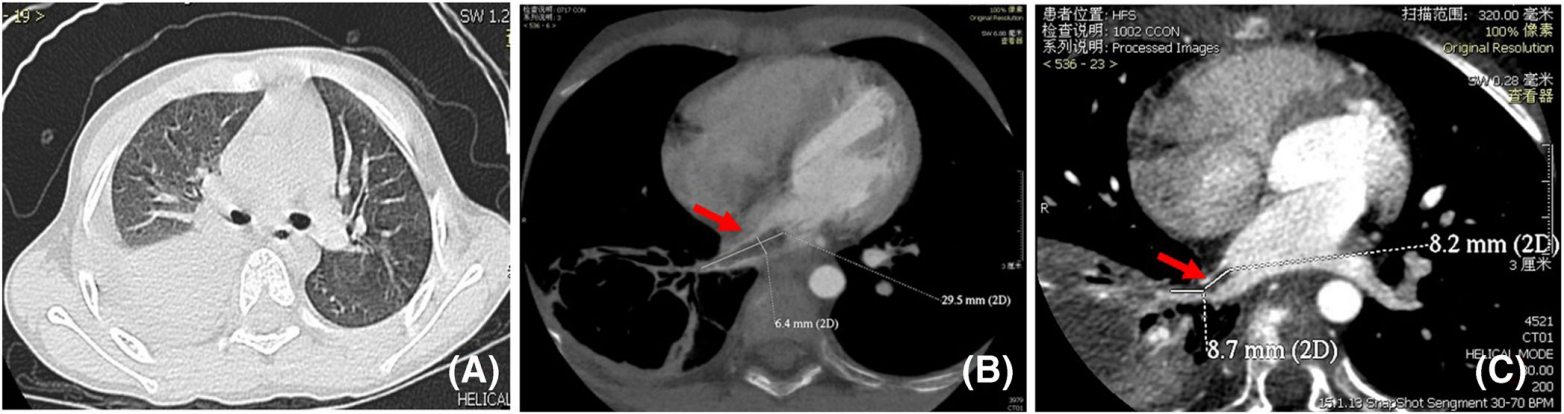
(A) CT showed consolidation of the right lower lung. (B) CTPA revealed a filling defect in the right pulmonary vein that extended to the left atrium; (C) the filling defect shrank after treatment

No specific symptoms or signs were demonstrated in patients with pulmonary vein thrombosis and cardiac thrombosis, and they were diagnosed using CTPA and echocardiography or enhanced MRI. There were five cases of pulmonary vein thrombosis; two of them only had pulmonary vein embolism. The two cases of pulmonary vein thrombosis were located in the right pulmonary vein; the thrombus extended to the left atrium in case 1 (Figure 1), and the other three cases were located in the right lower pulmonary vein and were complicated with pulmonary artery embolism.

**FIGURE 2:**
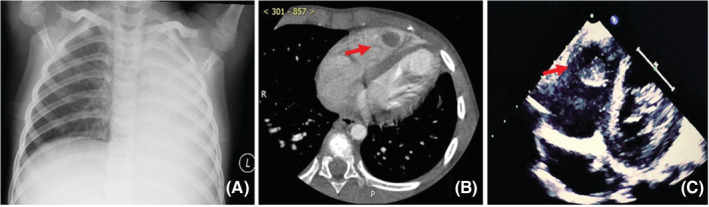
(A) Chest X‐ray at admission showed consolidation of the left lung; (B) the enhanced computed tomography taken after admission indicated a filling defect in the RV; (C) echocardiography was taken after admission, which showed a mass attached to the interventricular septum in the RV.

**FIGURE 3:**
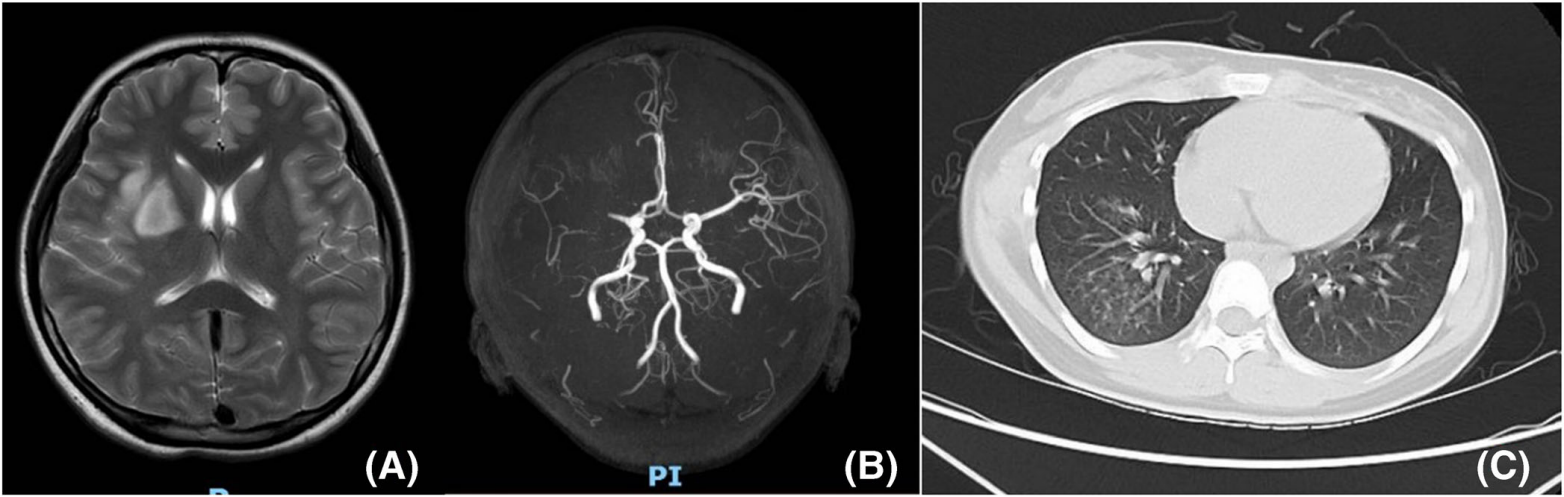
(A) MRI TW2 showed a high single change in the right brain; (B) MRA showed right MCA infarction; (C) the CT showed pneumonia of the right lower lung.

There were eight cases with cardiac thrombosis, including six in the ventricle and two in the atrium, and five cases with pulmonary vein thrombosis. No specific symptoms or signs were demonstrated in patients with cardiac thrombus and pulmonary vein thrombus. Case 2 was the first and only case of cardiac thrombosis diagnosed by pathology. A mass in the right ventricle was revealed in this 6‐year‐old boy diagnosed with severe *MPP* (Figure 2). On physical examination, the patient was weak but hemodynamically stable. Coagulation studies showed elevated D‐dimer. Although thrombosis is highly suspected, there was no obvious shrinkage of the mass after the antibiotic and the anticoagulant therapy of LMWH for 2 weeks. Hence, this mass was removed by surgery. The mass was diagnosed as a fibrin thrombus after a pathological examination. Longer observation periods were given for the subsequent cases. The thrombosis disappeared after anticoagulant therapy for 2 weeks to 3 months. Six cases of cerebral artery embolism had convulsion and hemiplegia. Case 3: an11‐year‐old girl was admitted because of fever, cough for 13 days, convulsions, and hemiplegia for 2 days. MRI showed the right middle cerebral artery embolism, and the pulmonary lesion was interstitial pneumonia of the right lower lung without consolidation (Figure 3). Two cases of cerebral sinus venous thrombosis (CSVT) only complained of headaches, whereas another developed cerebral hemorrhage secondary to sinus venous thrombosis and had headaches and hemiplegia.

The patient with multiple arterial embolisms of the celiac trunk, superior mesenteric artery, and splenic artery manifested abdominal pain, diarrhea, and vomiting.

Four cases of lower extremity DVT had symptoms of limb swelling and pain. Three cases were unilateral, and one case was bilateral.

Thirteen cases had thromboembolism of multiple anatomic sites as seen in Chart 1, mainly PE with thromboembolism of other sites (12 cases) and one case of multiple arterial embolisms.

### 3.4 Laboratory findings

At the time of admission due to MP pneumonia, white blood cell counts were normal or moderately elevated (mean, 11.40 ± 4.33 × 10^9^/L), Hemoglobin (mean, 115.12 ± 16.11 g/L), and platelet counts (319.65 ± 145.91 × 10^9^/L) were generally normal. The levels of C‐reaction protein, lactate dehydrogenase (LDH), and erythrocyte sedimentation rate (ESR) was all increased, with a mean value of 54.08 ± 52.27 g/L, 451.12 ± 218.76 U/L, and 43.40 ± 29.43 mm/h, respectively.

At the time of diagnosis of VTE, D‐dimer was elevated in all patients (median, 3.81; range, 0.34–48 ng/ml, normal 0–0.243 ng/ml). It was elevated in 81.6% of patients (40/49) with the value time increased (>10‐fold increase, >2.43 mg/dl). Blood coagulation tests showed normal PT (12.79 ± 1.75 s), aPTT (32.43 ± 6.34 s), and fibrinogen (3.81 ± 1.18 g/L) levels.

Some patients were screened for antiphospholipid (aPL) antibodies, including Lupus anticoagulant (LA), anti‐cardiolipin (aCL) antibodies, and anti‐beta2 glycoprotein I (aβ2GPI) antibodies. The results showed that LA was positive in 74.3% (26/35) of the cases, aCL‐IgM was positive in 66.7% (26/39) cases: aβ2GPI‐IgM was positive in 79.4% (27/34) of the cases. In most cases, the aPL antibodies turned negative in the majority of cases after 1–3 months of follow up, but there were three cases with positive LA at 6 months. Protein C and protein S were tested in 17 patients, with abnormal results in five of them: two with slightly lower protein C and protein S levels, and three with slightly lower protein S levels.

The case group was divided into two subgroups: The simple pulmonary artery and/or pulmonary vein thromboembolism group (*n* = 25) and the extra‐pulmonary thromboembolism group (*n* = 24). There was no significant difference in leukocyte, CRP, ESR, LDH, and D‐dimer values between the two groups. There was no significant difference in the positive rate of aPL between the two subgroups.

**FIGURE 4:**
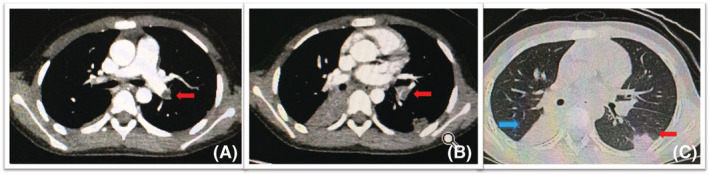
CTPA of Case 4: A shows truncation sign of left pulmonary artery trunk; B shows filling defect in left lower pulmonary artery; C shows atelectasis in the dorsal segment of right upper lung and subpleural wedge‐shaped high‐density shadow.

*Gene analysis* was performed on three patients with thromboembolism of multiple anatomic sites. Case 4, a 7‐years old boy, complained of swelling and pain in the left lower limb; chest pain, and dyspnea on the day after bronchoscopy under general anesthesia; DVT in the left lower limb and PE of the left pulmonary artery trunk were diagnosed (Figure 4). The lab test showed positive LA and aβ2GPI‐IgM, normal protein C, and protein S levels. Genetic analysis revealed a PROC gene variation (c.572‐574 del, p.193del). Case 5, a 13‐years old girl, had chest pain and dyspnea in the course of pneumonia. PE and splenic artery embolism were revealed by CTPA, and lab tests showed positive LA and aβ2GPI‐IgM, decreased protein C (36, normal 70–140), and protein S (49, normal 76–135) levels. Genetic analysis revealed a Factor V Leiden gene variation (c.4192A>T, p.Ser1398Cys) and the diagnosis of resistance to activated protein C. Case 6, an 11 years old, was diagnosed as DVT of bilateral limbs and pulmonary embolism, LA, and aβ2GPI‐IgM were positive. The whole exon gene sequencing analysis found no gene variation associated with inherited thrombophilia.

### 3.5 Treatment

Thrombolysis therapy (Recombinant human tissue plasminogen activator, rtPA) was given only to one patient in a local hospital. No massive hemorrhage occurred after rtPA was used. Lower molecular weight heparin (LMWH) was used for initial anticoagulant therapy in all 49 patients, except two patients with cerebral infarction (while in these two patients, low molecular dextrin was used). Warfarin was prescribed for follow‐up anticoagulant therapy in eight patients. The duration of anticoagulant therapy ranged from 1 month to 2.5 years. Anticoagulant therapy, in general, was well tolerated. Epistaxis and increased volume of menses were the main adverse events. No massive hemorrhage occurred.

### 3.6 Outcome

No deaths occurred in this group. The thromboembolism was mostly relieved at a 3‐month follow‐up. The prognosis was generally good; however, sequelae of hemiplegia occurred in one case of middle cerebral artery infarction. There was no recurrence of thromboembolism in this group till the follow‐up.

## 4 DISCUSSION

Thromboembolism is relatively rare in children and is mainly associated with critical illness, the presence of a central venous line (CVL), cancer, and congenital heart or vascular malformation,^8^, ^9^ However, recent reports have confirmed the correlation between infection and thromboembolism. Infection of different pathogens could cause thromboembolism, like a virus, as confirmed during the COVID‐19 pandemic.^10^ *M. pneumoniae* is an important pathogen of respiratory tract infections in children. We report a group of cases with thromboembolism complicated by MPP to summarize the clinical characteristics of the MPP extrapulmonary manifestations. From our case series report, we demonstrated that MPP is an important risk factor for thromboembolism, mainly in pulmonary vascular, but less in extra‐pulmonary.

Mechanisms proposed for extra‐pulmonary manifestations of MPP include direct invasion, cytotoxicity, and inflammation through the activation of immune system cells for cytokine production and immune response with the production of cross‐reactive antibodies by molecular mimicry, which may trigger autoimmune disorders.^11^ In our case series, pulmonary vascular thromboembolism was most common in 35 cases. The majority of pulmonary artery embolism cases were in situ pulmonary artery thrombosis (ISPAT), not classic thromboembolic PE (which often had co‐existing thromboses in the veins of either the lower extremity or at nonextremity sites),^12^ and pulmonary artery TE is significantly associated with necrotizing pneumonia. These indicated that the vasculitis caused by the direct invasion contributed to the local thromboembolism. The inflammatory factors, including the C‐reaction protein, lactate dehydrogenase, and erythrocyte sedimentation rate, were all increased, suggesting a systemic inflammatory response caused by mycoplasma pneumonia infection. There was no significant difference in inflammatory parameters between children with pulmonary vascular thromboembolism and those with thromboembolism at extrapulmonary sites, indicating that the systemic inflammatory process contributed to the thromboembolism at different anatomic sites.

Antiphospholipid antibodies (aPL), as commonly found in systemic lupus erythematosus, are associated with an increased risk of arterial and venous thrombosis.^13^ The appearance of these antibodies is reported in conjunction with various infections.^14^ Case reports of patients have indicated that aCL, LA, and b2 GPI can occur after infection with clinical consequences, not just as a transient non‐pathogenic process.^15^ Flateau et al. described how immune mediation might also play a part in the hypercoagulability seen in *M. pneumoniae* infection: aPL reacts against proteins that bind to phospholipids on plasma membranes, contributing to thrombosis.^16^ A previous study reported that mycoplasma pneumonia infection induces positive antiphospholipid antibodies without thrombosis.^17^ In our cases, the high positive rate of tested aPL (LA 74.3%, aCL‐IgM 66.7%, and aβ2GPI‐IgM 79.4%) suggested that they might actively contribute to hypercoagulability.

Because thromboembolism is often caused by more than one risk factor, other factors were evaluated except the MP infection because it was a retrospective study. There was no record of immobility during the treatment of pneumonia, and there were five patients with a history of bronchoscopy under general anesthesia before the onset of thromboembolism. In addition, although there was no family history of thromboembolism, inherited factors were tested in some patients. Five patients had abnormal protein C and/or protein S levels, but they did not meet the diagnostic criteria of congenital protein C or protein S deficiency. Gene analyses were performed on three patients with TE of multiple anatomic sites. PROC gene variation and Factor V Leiden gene variation were found in two patients, respectively, whereas no gene variation was associated with inherited thrombophilia. The detection of protein C and protein S is easily affected by many factors, such as age, sampling time, medication, infection, and type of gene variation. The cost of genetic analysis is high, so it remains controversial whether children with VTE benefit from IT screening.^18^

The D‐dimer represents the activation of coagulation and fibrinolysis systems. The D‐dimer level is one of the measures used in patients to detect thromboembolism. In addition, underlying diseases such as inflammation and surgery may trigger an increase in D‐dimer levels.^19^ Previous studies of the utility of D‐dimers for thromboembolism diagnosis in children have shown high sensitivity but a lack of specificity,^20^, ^21^ Some studies have reported that plasma D‐dimer levels significantly increase in children with acute MPP^22^ and are associated with NP.^23^ In our cases, the blood coagulation test showed increased levels of D‐dimer but no changes in PT and aPTT. D‐dimer was elevated in all patients with a median value of 3.81 ng/ml, which indicated it has a high sensitivity in predicting thromboembolism in MPP patients. However, it was difficult to determine a cut‐off value for the suspicion of thromboembolism in MPP in this study due to the small number.

The increased thromboembolism in pediatrics may result from more sensitive radiologic techniques to identify less clinically significant TEs or a higher suspicion index in physicians.^24^, ^25^ In our study group, more than half of PE patients without specific symptoms of thromboembolism had more CTPA contributing to this increase in diagnosis. For venous thrombosis, DVT of the lower limbs presented with swelling and pain in the lower limbs, whereas there were no specific symptoms in cardiac and pulmonary vein thrombosis. Cerebral artery and sinus venous thromboembolism symptoms were the most significant, whereas splenic infarction was asymptomatic. In addition to Kim's report of four cases of PE associated with macrolide‐resistant mycoplasma pneumonia,^26^ there are few discussions about the thromboembolism complications with macrolide‐resistant in previous literature reports. In this case, only five cases were tested for macrolide‐resistant, with three positive and two negatives. It is challenging to determine whether macrolide resistance is associated with thromboembolism complications because of the small number of cases. The imaging characteristics of pneumonia in this group were consolidation of the lung lobe or segment and pleural effusion. However, two cases also had interstitial changes (case 3 with MCA embolism and another case with both cerebral sinus venous thrombosis and PE). In our case group, all thromboembolism was diagnosed in the pneumonia patients caused by MP, but in a few reports, MP infection, without evidence of pneumonia, or respiratory failure, could also cause thromboembolism.^27^ Consolidation of the lung may be a risk factor for thromboembolism, but more case–control studies are needed to verify this. It is suggested that various forms of MP infection could cause thromboembolism complications.

Thrombolysis therapy (rtPA) was given only to one patient in a local hospital. No massive hemorrhage occurred after rtPA. LMWH was mainly used for initial anticoagulants, and Warfarin was prescribed for sequential anticoagulant therapy in eight patients. Anticoagulant therapy, in general, was well tolerated. There was no certain duration for the anticoagulant therapy. The duration in this group ranged from 1 month to 2.5 years, which was determined by the retest of the D‐dimer level, recovery of imaging, and the rechecked thrombophilia factors. No deaths occurred in this group. The prognosis was generally good, but sequelae of hemiplegia occurred in one case of middle cerebral artery infarction.

### 4.1 Limitations

This study is a retrospective study. The clinical data and laboratory examinations of some patients are incomplete, and it lacks a control group, which could exacerbate the difficulties in understanding the disease. However, the higher recognition of thromboembolism complication in MPP could help diagnose and manage such cases in the future.

### 4.2 Conclusion

Pulmonary arteriovenous thromboembolism is the most common thromboembolism complicated in MPP, and cerebral artery embolism and cardiac thrombosis are common in extrapulmonary thromboembolism. In the cases of MPP with thromboembolic complications, pulmonary consolidation with pleural effusion is the main characteristic. About two thirds of the cases are positive for antiphospholipid antibodies.

## CONFLICT OF INTEREST

The authors declare that they have no competing interests.

## ETHICS STATEMENT

The study was conducted following the Declaration of Helsinki and approved by the Ethics Committee at Beijing Children's Hospital, Capital University of Medical Science (protocol code [2022]‐E‐033‐R).

## AUTHOR CONTRIBUTIONS

All of the authors had access to the full dataset (including the statistical reports and tables) and took responsibility for the integrity and accuracy of the data analysis. The study was conceptualized by BPX, KLS, and LQC. The analysis and data collection were done by LQC, YJ, XYL, and JL. The initial draft of the manuscript was written by LQC. KLS reviewed and approved the final report. All authors read and approved the final manuscript.

## Data Availability

The data that support the findings of this study are available on request from the corresponding author. The data are not publicly available due to privacy or ethical restrictions.
